# Size-dependent cytotoxicity of silver nanoparticles in human lung cells: the role of cellular uptake, agglomeration and Ag release

**DOI:** 10.1186/1743-8977-11-11

**Published:** 2014-02-17

**Authors:** Anda R Gliga, Sara Skoglund, Inger Odnevall Wallinder, Bengt Fadeel, Hanna L Karlsson

**Affiliations:** 1 Division of Molecular Toxicology, Institute of Environmental Medicine, Karolinska Institutet, SE-171 77 Stockholm, Sweden; 2 KTH Royal Institute of Technology, Division of Surface and Corrosion Science, School of Chemical Science and Engineering, SE-100 44 Stockholm, Sweden

**Keywords:** Silver nanoparticles, BEAS-2B cells, Size-dependent toxicity, Cytotoxicity, Genotoxicity, Silver release

## Abstract

**Background:**

Silver nanoparticles (AgNPs) are currently one of the most manufactured nanomaterials. A wide range of toxicity studies have been performed on various AgNPs, but these studies report a high variation in toxicity and often lack proper particle characterization. The aim of this study was to investigate size- and coating-dependent toxicity of thoroughly characterized AgNPs following exposure of human lung cells and to explore the mechanisms of toxicity.

**Methods:**

BEAS-2B cells were exposed to citrate coated AgNPs of different primary particle sizes (10, 40 and 75 nm) as well as to 10 nm PVP coated and 50 nm uncoated AgNPs. The particle agglomeration in cell medium was investigated by photon cross correlation spectroscopy (PCCS); cell viability by LDH and Alamar Blue assay; ROS induction by DCFH-DA assay; genotoxicity by alkaline comet assay and γH_2_AX foci formation; uptake and intracellular localization by transmission electron microscopy (TEM); and cellular dose as well as Ag release by atomic absorption spectroscopy (AAS).

**Results:**

The results showed cytotoxicity only of the 10 nm particles independent of surface coating. In contrast, all AgNPs tested caused an increase in overall DNA damage after 24 h assessed by the comet assay, suggesting independent mechanisms for cytotoxicity and DNA damage. However, there was no γH_2_AX foci formation and no increased production of intracellular reactive oxygen species (ROS). The reasons for the higher toxicity of the 10 nm particles were explored by investigating particle agglomeration in cell medium, cellular uptake, intracellular localization and Ag release. Despite different agglomeration patterns, there was no evident difference in the uptake or intracellular localization of the citrate and PVP coated AgNPs. However, the 10 nm particles released significantly more Ag compared with all other AgNPs (approx. 24 wt% *vs*. 4–7 wt%) following 24 h in cell medium. The released fraction in cell medium did not induce any cytotoxicity, thus implying that intracellular Ag release was responsible for the toxicity.

**Conclusions:**

This study shows that small AgNPs (10 nm) are cytotoxic for human lung cells and that the toxicity observed is associated with the rate of intracellular Ag release, a ‘Trojan horse’ effect.

## Background

The past decades have witnessed increasingly rapid advances in the field of nanotechnology with the production of numerous engineered nanoparticles that bear outstanding optical, magnetic, catalytic and electrical properties [1]. Silver nanoparticles (AgNPs) are, largely due to their antimicrobial properties, the most commonly used engineered nanoparticles in commercialized products [2]. Approximately 320 tons of AgNPs are manufactured each year [3]. They are used in nanomedical devices, consumer products such as cosmetics, clothing, household products, room sprays and even in food products. Recently, AgNPs have gained attention for medical imaging and biosensing purposes [4,5]. However, human exposure is not new and AgNPs have a history of more than 100 years of use [3]. Inevitably, from the rapid increase in its manufacture and utilization follows an increased human (and environmental) exposure, whereas the potential toxicity has yet to be fully addressed.

The *in vitro* toxicity of AgNPs has been evaluated in a wide range of studies but there is still a lack of consistent and reliable data. This is a general concern in nanotoxicology and more research coherence is required for producing meaningful results [6]. In a recent review, Kim and Ryu [7] identified increased oxidative stress, apoptosis and genotoxicity to be the main *in vitro* outcomes upon exposure to AgNPs. The major drawback was that the investigated AgNPs were different in each study; *i.e.* manufactured in different ways, more or less purified, with various size distributions and coatings, tested on different cell lines under different cell culture conditions, and often without the use of reference materials. Moreover, there was in general a lack of thorough characterization of the AgNPs in cell medium. In all, with contradictory findings reported, there is at present no general agreement on the *in vitro* toxicity of AgNPs. A study by Hackenberg *et al*. [8] reported reduced cell viability at a AgNP dose of 10 μg/mL (1 h exposure, AgNPs <50nm) in human mesenchymal stem cells, whereas Samberg *et al*. [9] showed no toxicity for progenitor human adipose-derived stem cells up to 100 μg/mL (10 and 20 nm AgNPs for 24 h, and then differentiated for 14 days). Also, the stability and aging of AgNPs have been reported to be important for the toxicological outcome. Kittler *et al*. [10] showed a significant increase in toxicity following storage of AgNPs (in water) up to 6 months and this was correlated with the release of Ag ions. Ultimately, the synthesis method and the presence of residual contaminants could also account for the observed toxicity [11].

In addition to reported variations in cytotoxicity, there is a lack of consensus on the underlying mechanisms that drive the toxicity of AgNPs: the particles *per se*, the released Ag ionic species, or their combination. For example, Beer *et al*. [12] suggested that the cytotoxic effects of AgNPs, following exposure of A549 cells, were largely explained by released Ag ions. In a follow up study, the global gene expression profiling in the same cell line suggested that even though the responses to Ag ions and AgNPs were related as regards effects such as induction of metallothioneins, the AgNPs ultimately affected the cells in a more complex way (for instance by influencing expression of genes coding for cell cycle proteins) [13]. We recently showed that the cellular uptake of Ag was significantly higher when cells (A549 and BEAS-2B) were exposed to Ag as NPs rather than ions [14]. Thus, there is emerging evidence for the ‘Trojan horse’ hypothesis according to which the particle mediates the AgNPs uptake via endocytosis thereby increasing the intracellular bioavailability of Ag. Some previous studies have focused on investigating size dependent effects of AgNPs. However, whereas for example Liu *et al*. [15] reported that 5 nm AgNPs were more toxic compared with particles sized 20 and 50 nm, respectively, in four different cell lines (A549, HepG2, MCF-7 and CGC-7901), Kim *et al*. [16] showed an enhanced release of LDH in the presence of 100 nm sized AgNPs and reduced cell viability when compared to smaller sized particles (10 and 50 nm). In all, few studies exist in which size dependent effects and underlying mechanisms have been investigated using properly characterized AgNPs. The aim of this study was to investigate the toxicity of a panel of highly purified and well-characterized AgNPs with a specific focus on size- and coating-dependent effects, and to explore the mechanisms of toxicity. To this end, we used the BEAS-2B cell line, normal human bronchial epithelial cells (SV-40 immortalized) that are often used as a lung cell model.

## Results

### Characterization of a panel of AgNPs

**Figure 1:**
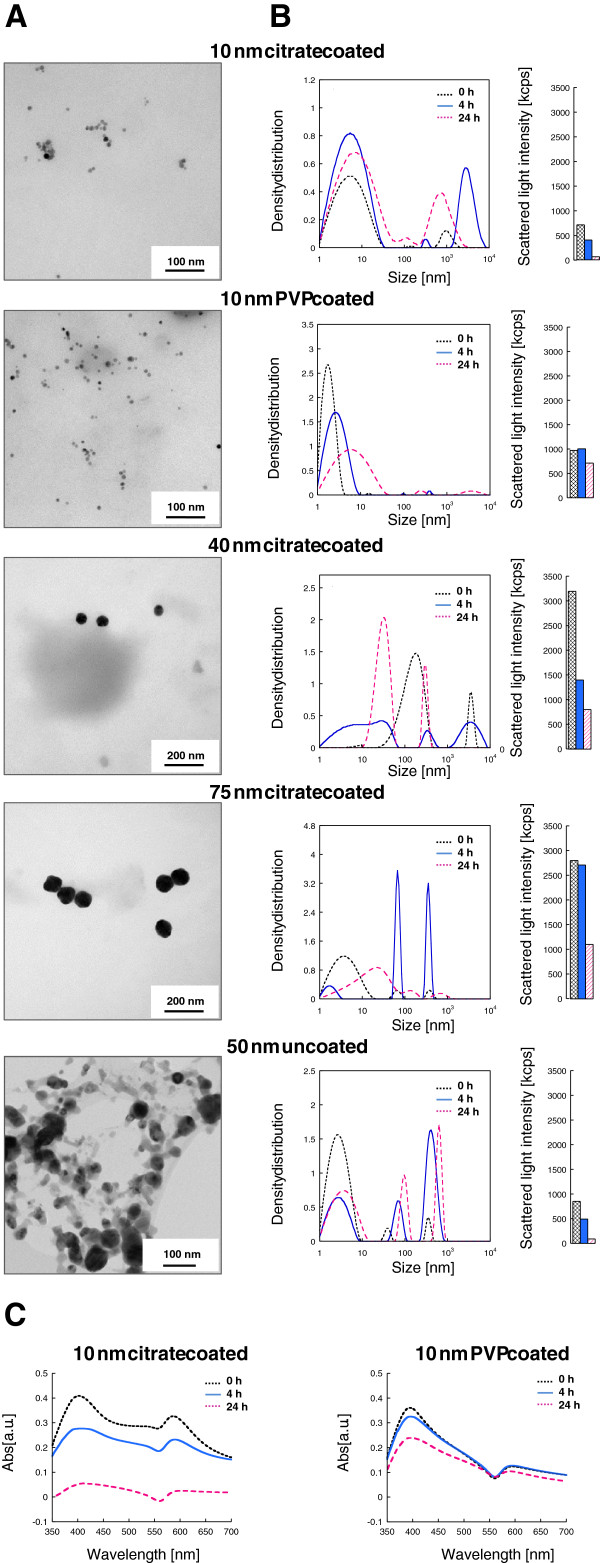
**AgNPs characterization by TEM (A), PCCS (B) and UV–vis (C).** TEM images of the AgNPs confirmed the primary size stated by the manufacturers **(A)**. The hydrodynamic size distribution was performed in cell medium (BEGM) using PCCS. Data is presented as density distribution by volume together with the corresponding scattered light intensity **(B)**. All the AgNPs agglomerated in cell medium as seen by the size and position of the density distribution peaks. All but the 10 nm PVP coated AgNPs sedimented significantly over time as indicated by the reduction of the scattered light intensity. The UV–vis spectra of the 10 nm citrate (**C** right) and 10 nm PVP (**C** left) coated AgNPs indicated particle sedimentation over time as seen from the 400 nm peak reduction.

NPs from commercial sources were evaluated for their primary size by TEM and for their agglomeration in cell medium by photon cross correlation spectroscopy (PCCS) and ultraviolet–visible (UV–vis) spectroscopy. Representative TEM pictures of the AgNPs are shown in Figure 1A. The TEM images of the coated AgNPs dispersed in water confirmed the primary particle size stated by the manufacturers. The uncoated particles had a heterogeneous distribution within the range of 40 to 200 nm, most of them being around 50 nm.

Changes in hydrodynamic size distribution and state of agglomeration of all AgNPs monitored directly after dispersion (0 h) and after 4 h as well as 24 h in cell medium (BEGM) are presented as density distribution (by volume) with size in Figure 1B together with the changes in scattered light intensities for the corresponding time points. The particle size distribution by volume (d_0.1,_ d_0.5,_ d_0.9_) is included as an Additional file 1: Table S1. When interpreting the data it should be noted that the intensity of the scattered light increases non-linearly with increasing particle size, and that two processes hence occur simultaneously, agglomeration (increased intensity) and sedimentation (reduced intensity). Furthermore, there is a risk of overestimation of the proportion of small particles in the size distributions curves due to rotational diffusion that can take place for non-spherical particles and give rise to a peak at small particle sizes (<10 nm) [17]. The results showed that particle agglomeration as well as sedimentation, indicated by a reduction of the scattered light, was evident for all AgNPs with time but there was a clear difference between the citrate and PVP coatings. The 10 nm citrate coated AgNPs initially showed a trimodal size distribution, with peaks centered approximately at 10, 100 and 1000 nm. The two larger modes refer to agglomerates, also seen from the TEM investigation, and are expected due to the high ionic strength in the cell medium [18]. The peak at 10 nm refers both to particles of this size and to the rotational diffusion effect, as described above. After 4 h, the two larger modes were shifted towards larger sizes indicating further agglomeration. However, after 24 h the size distribution was similar as the initial observations with smaller size particle/agglomerate distributions. An evident reduction in scattered light intensity with time indicates fewer particles in solution and thus, the discrepancy between 4 h and 24 h is predominantly explained by sedimentation of the largest particles from which follows a reduced intensity and reduced size distribution of particles still in solution. The 10 nm citrate coated AgNPs agglomerated directly after dispersion (0 h), were less stable with time in cell medium, and sedimented to a larger extent when compared with the 10 nm PVP coated AgNPs. The latter particles showed mostly small particles (<10 nm) even after 24 h, and only a low amount of agglomerates of larger sizes. Also the scattered light intensity was relatively stable with time, indicating a higher stability. The observed differences in agglomeration and sedimentation behavior of the citrate and PVP coated 10 nm particles were further confirmed by UV–vis measurements (Figure 1C), showing a reduced absorbance with time for the citrate and PVP coated particles due to sedimentation. The rate of sedimentation was higher for the citrate coated particles as compared to the PVP coated AgNPs, in agreement with the PCCS findings. Also there was a slight broadening of the peaks with time, explained by the formation of larger agglomerates [19].

The freshly prepared 40 nm citrate coated AgNPs had a trimodal size distribution, with the peaks broadening out with time up to 4 h. The proportion of the peak of the largest agglomerates (1000–10000 nm) was reduced and vanished after 24 h. Similar to findings for the 10 nm citrate coated particles, the intensity of the scattered light was reduced at the same time as the size distribution became bimodal and more narrow again due to further agglomeration of the smallest particles and sedimentation of the larger agglomerates. The 75 nm citrate coated AgNPs initially showed a trimodal distribution and an increased agglomeration with time. After 24 h the larger agglomerates sedimented and the smaller particles became more agglomerated. The uncoated AgNPs also agglomerated with time but, after 24 h there were no large agglomerates (>200 nm) in solution. This might be explained by a higher rate of agglomeration for the uncoated particles, resulting in large agglomerates that due to sedimentation were not detected. The observed presence of particles sized less than <10 nm has been verified for the same batch of AgNPs elsewhere [20].

### 10 nm AgNPs are cytotoxic for human lung cells

**Figure 2:**
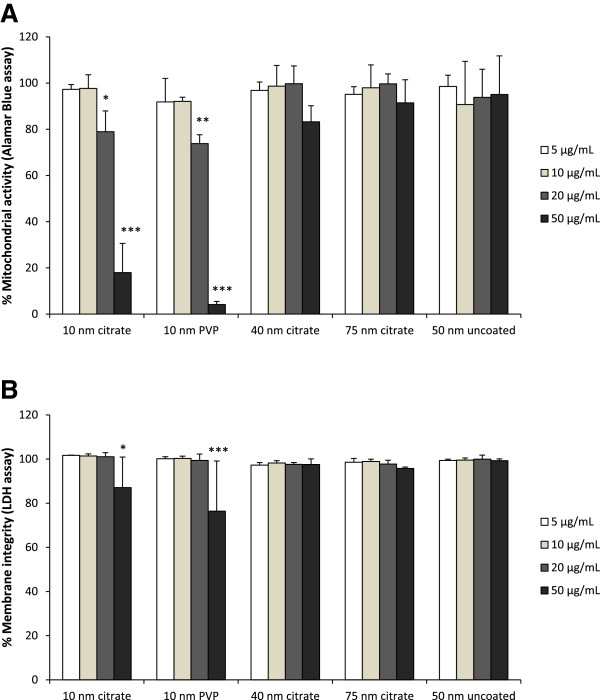
**Cell viability of BEAS-2B cells after exposure to AgNPs.** Cell viability of BEAS-2B cells after 24 h exposure to AgNPs (5–50 μg/mL) was assessed using Alamar Blue assay **(A)** and LDH assay **(B)**. Results are expressed as % mitochondrial activity for the Alamar Blue assay and % membrane integrity for the LDH assay. Results are presented as mean ± standard deviation of 3 independent experiments. Significant results as compared to the control are marked with asterisks (* for P-value < 0.05, ** for P-value < 0.01, *** for P-value < 0.001).

Cytotoxicity of AgNPs was evaluated using two different assays: Alamar Blue (AB) and Lactate dehydrogenase (LDH) assays. The AB assay was used to assess cell viability and cell proliferation and is based on the reduction potential of metabolically active cells. The read out gives indications on overall mitochondrial activity after short exposure time periods (4 h) and is also a measure of cell proliferation at longer exposure times that allow for cell division (24 h). BEAS-2B cells were exposed to AgNPs of different doses (5–50 μg/mL) for 4 and 24 h. After 4 h, no significant signs of toxicity were observed for any of the AgNPs up to the highest dose tested (Additional file 2: Figure S1). Significant cell toxicity was only evident for the 10 nm citrate coated (p ≤ 0.05) and the 10 nm PVP coated (p ≤ 0.01) AgNPs after 24 h for their highest doses (20 and 50 μg/mL). No significant alterations of the mitochondrial activity of the BEAS-2B cells were observed for any of the lower doses (5 and 10 μg/mL) or the other AgNPs (Figure 2A). The interference of the AgNPs with the AB assay was tested in an acellular system and found to be non-significant (Additional file 3: Figure S2).

The LDH assay is a cytotoxicity assay that measures membrane damage by quantifying the amount of LDH released from the cytoplasm. BEAS-2B cells were exposed to AgNPs (5–50 μg/mL) for 4 and 24 h. No significant toxicity was observed after 4 h for any of the AgNPs (Additional file 2: Figure S1). However, significant toxicity was observed after 24 h for the 10 nm citrate coated (p ≤ 0.05) and the 10 nm PVP coated (p ≤ 0.0001) AgNPs at the highest dose (50 μg/mL). None of the larger sized AgNPs altered the cell viability (Figure 2B).

Some AgNPs have been shown to interact with the LDH assay via enzyme inhibition or binding [21,22]. To investigate this issue we incubated AgNPs with cell lysates and detected the LDH activity after 0, 4 and 24 h (Additional file 3: Figure S3). The reduction in enzyme activity was most pronounced for the 10 nm AgNPs, especially for the citrate coated particles, and occurred in a time and dose dependent manner. The enzyme inhibition is likely correlated with the Ag release since Ag ions have been shown to inhibit the catalytic activity of LDH enzyme [23]. Therefore, LDH results should be interpreted with caution and the possibility of false negative results be considered (as a results of enzyme inhibition in the analyzed supernatant), especially for particles with low stability that release Ag ions.

### AgNPs induce DNA damage in human lung cells

The potential of AgNPs to induce DNA damage was investigated with two different assays: alkaline comet assay that gives indication on the overall DNA damage (DNA strand breaks and alkali labile sites) and γH_2_AX foci formation, which is mainly a marker of DNA double strand breaks.

**Figure 3:**
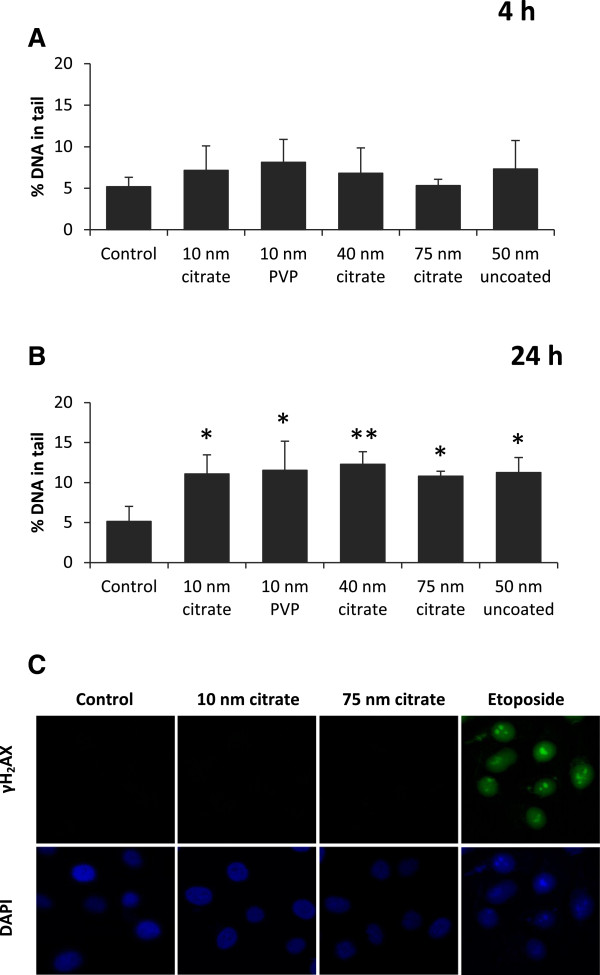
**Genotoxicity of AgNPs in BEAS-2B cells.** Comet assay was performed after 4 h **(A)** and 24 h **(B)** exposure to AgNPs. The induction of DNA damage was evaluated after 4 and 24 h incubation with 10 μg/mL AgNPs. DNA damage was expressed as % DNA in tail. Results are presented as mean ± standard deviation of at least 3 independent experiments. Significant results as compared to the control are marked with asterisks (* for P-value < 0.05, ** for P-value < 0.01). Immunocytochemistry of γH_2_AX foci in BEAS-2B cells was performed after exposure to AgNPs for 24 h **(C)**. Cells were treated for 24 h with 10 μg/mL AgNPs (10 nm and 75 nm citrate coated) and etoposide 10 μM (positive control). After exposure, cells were fixed and stained for γH_2_AX foci (FITC conjugated anti-phospho-histone H_2_AX-Ser 139 antibody, green) and nucleus (DAPI, blue). Pictures were taken with a confocal laser scanning microscope, 40X, oil objective. Images are representative for 3 independent experiments.

The alkaline comet assay was used to determine the DNA damage associated with exposure to non-cytotoxic concentrations (10 μg/mL) of AgNPs in BEAS-2B cells. No significant increase in the percentage of DNA in the comet tail was observed after 4 h exposure to any of the AgNPs (Figure 3A). However, a statistically significant increase in overall DNA damage was observed after 24 h for all AgNPs, independent of size and coating (Figure 3B).

The γH_2_AX foci formation was assessed by immunocytochemistry in BEAS-2B cells under the same conditions as for the comet assay, *i.e.* 4 and 24 h exposure to 10 μg/mL AgNPs. All fluorescent stainings were negative for γH_2_AX both after 4 h (data not shown) and 24 h. Fluorescence images are shown in Figure 3C for two of the investigated particles, 10 nm and 75 nm citrate coated AgNPs. Etoposide, a topoisomerase inhibitor, was used as a positive control. The results show that none of the AgNPs induced DNA double strand breaks in BEAS-2B cells under given test conditions.

### No cellular ROS increase upon exposure to AgNPs

**Figure 4:**
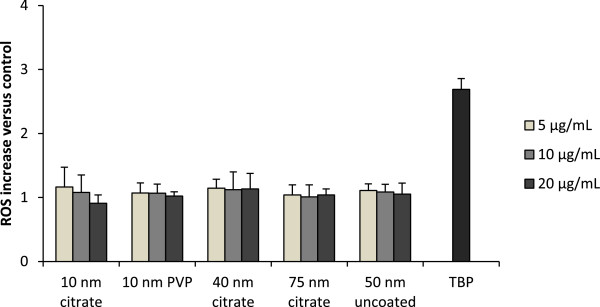
**ROS levels in BEAS-2B cells after exposure to AgNPs.** ROS formation after exposure to AgNPs was investigated using the DCFH-DA assay. Cells were incubated with AgNPs (5, 10, 20 μg/mL) for 24 h and then with 20 μM DCF-DA probe for 40 min. Readings (Ex485 nm/Em535 nm) were performed every 5 min over 30 min. Tert-butyl hydroperoxide (TBP, 15 μM) was used as positive control. ROS increase was calculated as mean slope per min and normalized to the unexposed control. Results are presented as mean ± standard deviation of 4 independent experiments.

The kinetics of intracellular ROS formation after exposure of BEAS-2B cells to AgNPs was measured using the dichlorodihydrofluorescein diacetate (DCFH-DA) assay. The DCFH-DA probe can detect cytosolic radicals such as hydroxyl, peroxyl, alkoxyl, nitrate and carbonate, but is not able to pass organelle membranes [24]. None of the AgNPs induced any significant ROS increase after 24 h, at doses up to 20 μg/mL (Figure 4). The positive control, tert-butyl hydroperoxide, induced a 2.8-fold increase compared with unexposed cells. No increased ROS generation was observed during the first 4 h of exposure (Additional file 4: Figure S4).

### AgNPs are readily taken up by human lung cells via active mechanisms

**Figure 5:**
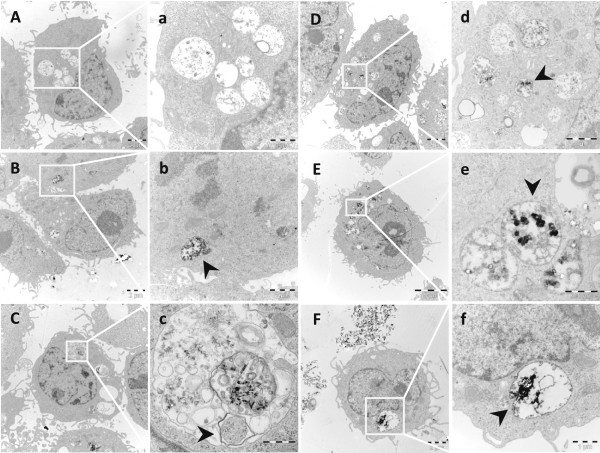
**Intracellular localization of AgNPs in BEAS-2B cells.** Intracellular localization of AgNPs was investigated by TEM **(A-F** with corresponding inserts **a-f**).TEM images of untreated BEAS-2B cells show no morphological modifications **(A, a)**. After 24 h exposure to 10 μg/mL 10 nm citrate coated **(B, b)**, 10 nm PVP coated **(C, c)**, 40 nm citrate coated **(D, d)**, 75 nm citrate coated **(E, e)**, and 50 nm uncoated **(F, f)** AgNPs, particles were taken up and contained mainly within membrane-bound structures **(b, d, e, f)**. Vesicular structures consistent with autophagy were detected for the 10 nm PVP coated AgNPs **(c)**.

We next investigated whether the differences in cytotoxicity could be explained by differences in cellular uptake or intracellular localization. Intracellular particle localization in BEAS-2B cells after exposure to 10 μg/mL AgNPs was investigated using TEM imaging. After 4 h exposure, AgNPs were taken up and were localized mainly within membrane-bound structures. No clear differences were observed between the different AgNPs in terms of uptake or intracellular localization. The corresponding TEM pictures are presented in the Additional file 5: Figure S5. After 24 h, all AgNPs were still mainly confined in membrane-bound structures (Figure 5). Moreover, cellular morphological changes suggestive of autophagy were observed for the 10 nm PVP coated AgNPs (Figure 5c). There were no signs of nuclear localization for any of the particles.

**Figure 6:**
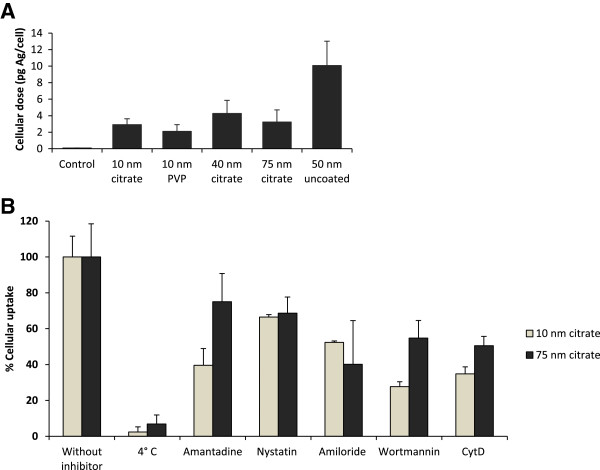
**Uptake of AgNPs by BEAS-2B cells.** The cellular Ag dose was quantified by AAS **(A)**. BEAS-2B cells were exposed to 10 μg/mL AgNPs for 4 h and the total cellular Ag content was analyzed by AAS. The Ag dose was expressed as pg per cell. Results are presented as mean ± standard deviation of 2 replicates. The uptake mechanisms were investigated using pharmacological inhibitors and 4°C exposure **(B)**. BEAS-2B cells were pre-incubated with different pharmacological inhibitors at 37°C (clathrin-mediated endocytosis: amantadine 200 μM for 30 min, caveolin/lipid raft mediated endocytosis: nystatin 25 μM for 30 min, macropinocytosis: amiloride-HCl 100 μM for 30 min, general fluid-phase endocytosis: wortmannin 400 nM for 30 min, actin-dependent phagocytosis: cytochalasin D 1 μM for 1 h). For energy dependent inhibition of uptake the cells were pre-incubated at 4°C for 30 min. Following the pre-incubations, cells were exposed to 10 μg/mL 10 nm citrate coated or 75 nm citrate coated AgNPs for 2 h in the presence of the inhibitors or at 4°C. The total Ag content was determined using AAS. The results are expressed as % of the corresponding controls and presented as mean ± standard deviation of 2 replicates.

The cellular dose (*i.e.* cellular uptake as well as AgNPs tightly attached to the cell membrane) of AgNPs in BEAS-2B cells was quantified using AAS analysis. These measurements resulted in an average Ag concentration per cell in the range of 2.1 – 10 pg after 4 h (Figure 6A). The results indicated the highest uptake (10 pg/cell) for the 50 nm uncoated AgNPs. There was no major difference between the PVP and citrate coated particles and no obvious size dependent uptake; the 10 nm and 75 nm citrate coated AgNPs showed similar cellular concentrations (2.9 and 3.2 pg/cell). When the data was converted to percentage uptake from the total added Ag the results were in the range of 3.2 and 12.1%.

The uptake mechanisms were addressed by using pharmacologic inhibitors of different endocytic pathways together with experiments performed at 4°C in which energy dependent uptake is stalled. We selected the 10 nm and 75 nm citrate coated AgNPs to identify a possible size-dependent difference in the uptake mechanisms. As shown in Figure 6B, both 10 nm and 75 nm citrate coated AgNPs were taken up by active mechanisms as evident by a negligible uptake at 4°C (2.4% and 6.8%). Actin dependent pathways were involved in the internalization of both particles as observed by the cytochalasin D inhibition (35% uptake for the 10 nm and 51% uptake for the 75 nm AgNPs). Overall the uptake was a combination of active mechanisms (clathrin, caveolin/lipid raft, macropinocytosis and phagocytosis) as indicated by the decreased uptake following treatment with the additional pharmacological inhibitors (amantadine, nystatin, amiloride and wortmannin).

### Small AgNPs release more Ag in biological medium

**Figure 7:**
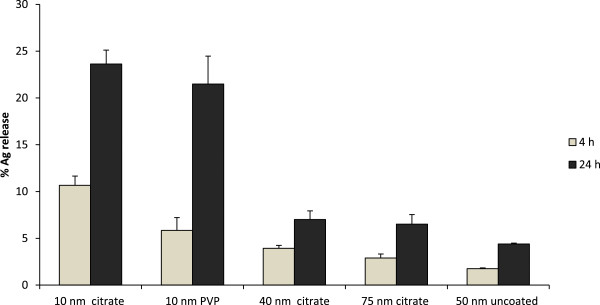
**Quantification of the Ag release in cell medium.** The amount of released Ag in cell medium (BEGM) after 4 and 24 h at 37°C was quantified by means of AAS and expressed as percentage from the total added Ag (10 μg/mL). Results are presented as mean ± standard deviation of 2 replicates.

The amount of released Ag present in solution (non-precipitated) from the AgNPs after 4 and 24 h incubation in cell medium (BEGM) is presented in Figure 7 in relation to the total amount of added AgNPs (10 μg/mL). The released amount of Ag in solution increased with time for all particles. The 10 nm citrate coated AgNPs revealed a higher Ag release in cell medium (10.6%) after 4 h compared with the 10 nm PVP coated AgNPs (5.8%). This discrepancy is related to differences in capping agent stability, as discussed below. However, after 24 h the release was more similar, 23.6% and 21.5% for the 10 nm citrate and PVP coated AgNPs, respectively. The 40 nm and 75 nm citrate coated AgNPs showed a relatively similar Ag release (3.9% and 2.9% at 4 h; 7% and 6.5% after 24 h). Overall the 50 nm uncoated AgNPs showed the lowest released fraction, (1.8% at 4 h and 4.4% at 24 h), likely related to their lower particle stability and hence a more rapid formation of larger agglomerates that sediment. As a result, the exposed surface area will be reduced thus slowing down dissolution kinetics. The total amount of Ag released in solution may, however, be underestimated due to complexation processes between released Ag and cell medium components and concomitant precipitation.

We then attempted to mimic the intracellular behavior of AgNPs by investigating the Ag release in ALF (artificial lysosomal fluid) of pH 4.5. As presented in Additional file 6: Figure S6, the overall amount of released Ag present in solution was very low (less than 2%), hence considerably lower than corresponding measurements in cell medium. This is related to the lack of stability and pronounced sedimentation of AgNPs in this fluid [25] and complexation of released Ag ionic species (*e.g.* AgCl). These findings are in agreement with a study by Stebounova *et al*. [25] who measured negligible released quantities of Ag in solution from AgNPs in two simulated biological fluids, artificial lysosomal fluid and artificial interstitial fluid.

**Figure 8:**
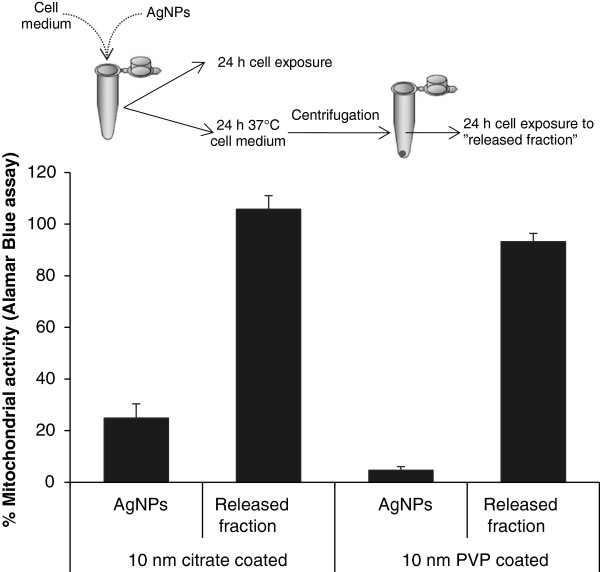
**Cytotoxicity of the extracellular release Ag fraction.** The Ag released fraction in cell medium was obtained by incubating 50 μg/mL AgNPs in BEGM at 37°C for 24 h. BEAS-2B cells were incubated with the respective supernatants for 24 h and cytotoxicity was measured with the Alamar Blue assay. Results are presented as mean ± standard deviation of 2 independent experiments.

In order to investigate whether the released Ag ionic species could account for the observed toxicity, the BEAS-2B cells were exposed for 24 h to the extracted “released Ag fraction”, *i.e.* the supernatants collected after 24 h incubation of 10 nm citrate and PVP coated AgNPs dispersions (50 μg/mL) in cell medium (Figure 8). However, there were no signs of toxicity as indicated by the AB assay, suggesting that the toxic effects observed after 24 h were not related to extracellular Ag release in cell medium.

## Discussion

The toxicity of AgNPs to eukaryotic cells, bacteria and multicellular organisms has been investigated in a number of studies, most of which overlook fundamental issues. For instance, not all studies indicated whether the nanoparticles were purified after synthesis or not, and many studies failed to describe the behavior of nanoparticles in the given biological media [26,27]. The purpose of this study was to investigate the toxicity of a panel of highly purified and well-characterized AgNPs with a specific focus on size- and coating-dependent effects, and to explore the mechanisms of possible differences in toxicity.

In the present study we used exposure concentrations in the range of 5–50 μg/mL, primarily based on previous studies of Ag nanoparticles and eukaryotic cells [26]. This may be related to a possible human exposure by using exposure data from a AgNPs manufacturing facility (maximum level 289 μg/m^3^[28]), and by applying the same assumptions and calculations as in the study by Wang *et al.*[29]. A concentration of 10 μg/mL would then approximately correspond to the total cellular deposition following 74 working weeks (8 h per day, 5 days per week). Thus, the doses used should be considered high but likely possible to be reached following years of exposure, or after acute accidental exposure.

The results showed a clear size dependent toxicity for the tested AgNPs since only the 10 nm AgNPs were cytotoxic for the BEAS-2B cells starting at doses of 20 μg/mL in the Alamar Blue assay. There was, however, no difference in toxicity between the 10 nm citrate and 10 nm PVP coated AgNPs, suggesting that the size rather than the capping agent was the property that triggered toxicity. Other studies have also reported higher toxicity for smaller compared to larger sized AgNPs. For example, Carlson *et al.*[30] showed an increased ROS generation for 15 nm hydrocarbon coated AgNPs as compared to 55 nm, which also correlated with decreased cell viability in macrophages. Furthermore, Liu *et al.*[15] found that 5 nm AgNPs were more toxic than 20 and 50 nm AgNPs in four cell lines (A549, HepG2, MCF-7, SGC-7901). Using the same kind of AgNPs as in the present study (10 nm PVP coated), George *et al.*[31] reported approximately 35% cytotoxicity following exposure of fish gill cells to doses of 25 μg/mL; thus, a very similar extent of cytotoxicity as in the present study, and no cytotoxicity for the 40 nm. Recently also Wang *et al.*[29] showed that 20 nm citrate and PVP coated AgNPs induced more cellular toxicity than larger particles (110 nm) and furthermore that the citrate coated 20 nm generated acute neutrophilic inflammation in the lungs of exposed mice to a much higher extent when compared to the larger ones.

In order to explore the genotoxicity of AgNPs in lung cells we used the alkaline version of the comet assay and γH_2_AX foci induction. In contrast to the size-dependent effect on cell viability, we found that all tested AgNPs induced DNA damage after 24 h as reported by the comet assay, but without γH_2_AX induction. There were, however, no signs of DNA damage at earlier time points (4 h) suggesting indirect genotoxic mechanisms that take more time to occur. The effect on cell viability and the DNA damage may potentially be explained by ROS generation [32]. However, we could not provide any evidence of intracellular ROS production preceding (geno)toxicity, thus contradicting many other published *in vitro* studies [7]. The comet assay is a highly sensitive method and widely applied in nanotoxicological studies [33], but it gives limited mechanistic insight. Thus, the more precise mechanism of genotoxicity warrants further investigation. One hypothetical explanation for the detected DNA damage could be the interaction of the particles with the DNA repair pathways. Such interactions have been previously reported for AgNPs *e.g.* reduction of the formamidopyrimidine DNA glycosylase activity [34] and down regulation of genes involved in DNA damage response/repair system (XRCC1 and 3, FEN1, RAD51C, RPA1) [35].

Next we investigated the mechanisms behind the observed size-dependent cytotoxicity by analysis of the cellular uptake and uptake mechanisms, intracellular localization, agglomeration and the released Ag fraction in cell medium. The TEM images showed that all AgNPs were mainly localized within membrane-bound structures. No AgNPs were detected in the cell nuclei, although nuclear presence has previously been reported for BEAS-2B cells (43–260 nm AgNPs) [36], U521 cells (6–20 nm AgNPs) [37], HaCaT cells (30 nm AgNPs) [38] and hMSC (AgNPs <50 nm) [8]. Multi-lamellar structures consistent with autophagy were observed for the 10 nm sized AgNPs. Induction of autophagy has been reported for several engineered nanoparticles, including AgNPs and Ag nanowires [37,39], and may represent a common cellular response to nanoparticles. In general, differences in the intracellular localization of the particles could not explain observed differences in toxicity. Moreover, when comparing the total cellular Ag content, determined by AAS, we could not detect a higher cellular dose of the most cytotoxic NPs, the 10 nm particles. Thus, the intracellular dose, that often is regarded to be of importance for toxicity, could not explain the higher toxicity of the 10 nm AgNPs. The total uptake was around 2–4 pg/cell for the coated and somewhat higher (approx. 10 pg/cell) for the uncoated AgNPs, in agreement with our previous studies of cellular uptake of AgNPs in BEAS-2B and A549 cells [14]. Observed findings are also within the same range as reported in a recent study on HepG2 cells with 6.8 pg Ag/cell following exposure to 10 μg/mL AgNPs for 24 h [40]. Interestingly, the same study further attempted to distinguish between AgNPs and Ag ions in the cells by using Triton-X 114-based cloud point extraction of the cell lysates. The authors concluded that approximately 10% of the total amount of Ag within the cells existed as Ag ions. Since this value was higher than the corresponding fraction of Ag ions prior to exposure (5.2%), they argued that transformation of AgNPs to Ag ions could have taken place intracellularly.

In the present study we carefully addressed time-dependent changes in agglomeration, an aspect often completely overlooked in studies within the field of nanotoxicology. There are several factors that should be taken into account when evaluating the agglomeration of the different particles. For example, it is well known that the intensity as measured using light scattering techniques increases with particles size in a non-linear manner, and at the same time sedimentation reduces the intensity thus making the interpretation non-trivial. Clearly, however, there was an evident difference in stability between the citrate and PVP coated 10 nm particles. This could be explained by the more rapid displacement of the electrostatically weakly bound citrate with medium components (*e.g.* aminoacids), triggered by the high ionic strength of the medium, when compared to the non-charged larger PVP polymer capping agent. A more rapid breakdown of the stabilizing coating will evidently affect the stability of the particles. The lower stability of the citrate coating also resulted in higher Ag release compared with the PVP coated Ag NPs in cell medium after 4 h. However, observed differences in agglomeration did not translate to differences in Ag release or toxicity after 24 h. This is perfectly in line with the recent study by Wang *et al.*[29] showing higher Ag release in BEGM media from 20 nm citrate coated Ag nanoparticles when compared to PVP coated particles at 6 h, followed by a very similar release at 24 h. Also, in accordance with our results, they report higher Ag release and toxicity from the smaller (20 nm) compared to the larger (110 nm) Ag nanoparticles. In all, the primary particle size seems to be more important than the size of the agglomerates for Ag release [41] and, according to the present study, for toxicity as well.

Proteins in the cell medium are known to be important for the stabilization of citrate coated AgNPs via the formation of a protein corona [42]. Therefore the low protein content of our working medium (serum free) could partially explain the agglomeration of the citrate coated particles upon dispersion. Ultimately the protein corona may play a role in the cellular uptake. Monteiro-Riviere *et al.*[43] recently showed that pre-incubation of citrate coated Ag nanoparticles with different proteins (albumin, IgG, transferrin) reduced the cellular uptake for both 20 nm and 110 nm particles. Yet, the similar behavior of the different sized nanoparticles used in this study together with the low protein content in the working cell medium, suggest that the protein corona is unlikely to explain the observed differences in toxicity. Differences in nanoparticle agglomeration affect sedimentation and may ultimately result in changes in the exposure doses and uptake rates [44]. However, the uptake of the 10 nm citrate and 10 nm PVP coated AgNPs was similar (2.9 pg/cell and 2.1 pg/cell) and in the same range as the 75 nm citrate coated AgNPs (3.2 pg/cell). Next we explored the uptake mechanisms for the 10 and 75 nm citrate coated AgNPs and found that both particles were internalized by active mechanisms as shown by the negligible uptake at 4°C. A combination of different active pathways was involved for both particles as previously shown for AgNPs [45] as well as other nanomaterials *e.g.* quantum dots [46]. Thus, while we acknowledge the importance of agglomeration for particle stability, and the fact that this, as well as the protein corona can affect cellular uptake, metal release and toxicity, it appears not to play a major role in the toxicity observed for the 10 nm citrate and 10 nm PVP coated particles.

The main difference between the AgNPs in our study was the released amount of Ag in cell medium, which was significantly higher for the 10 nm AgNPs. One explanation for this is obviously the increased surface area and increased particle number (196 fold increase for 10 nm *vs*. 40 nm particles and 1140 fold increase for 10 nm *vs.* 75 nm particles) for the same mass/volume dose. This is in line with previous reports showing that the release of Ag is directly related to the total surface of the particles as well as the composition of the experimental media [47]. Ag release has previously been reported to increase with smaller particle size in a non-linear manner [48], thus explaining the much higher release from the 10 nm particles when compared to the other sizes. To further explore the role of the released Ag, we also investigated the toxicity of the “released fraction” (*i.e*. the supernatant of centrifuged particles after incubation in cell medium for 24 h thus likely containing various Ag-complexes). However, no effect on cell viability was observed after incubating BEAS-2B cells with this fraction and we therefore concluded that the extracellular release and presence of ionic species in cell medium could not account for the observed differences in toxicity. We thus posit that the size dependent toxicity relates to the *intracellular* release of Ag ions. When we attempted to mimic one intracellular compartment, the lysosome, by using artificial lysosomal fluid (ALF), very little release was observed (Additional file 6: Figure S6). This is explained by the severe agglomeration that takes place in this solution due to the very high ionic strength [25] since low pH (in low ionic strength solutions) is known to cause higher Ag release [48]. In addition, ALF does not contain any proteins that can serve to stabilize the particles and we conclude that mimicking various intracellular compartments is challenging. Previous studies have shown that Ag ions interfere with cellular functions by interacting with the thiol and amino groups of biomolecules [26], thus providing an explanation for the toxicity. Ag release has also been reported to govern the toxicity of AgNPs towards bacteria, where the particles act as a vehicle for Ag delivery [49]. In the same study the antibacterial effect was hindered under anaerobic conditions (that prevent oxidation and Ag release) [49]. Moreover, AgNPs with higher Ag release were shown to be more toxic in *Caenorhabditis elegans*[50]. In all, this suggests that AgNPs may change the transport rate of Ag ions into cells and organisms and that subsequently released Ag ions exert the detrimental effects.

## Conclusion

The present study addresses aspects that often are overlooked in nanotoxicology studies such as careful time-dependent characterization of agglomeration and ion release. The study clearly shows size-dependent cytotoxicity of AgNPs since only the 10 nm particles affected the cell viability of human lung cells. Despite differences in agglomeration of the citrate and PVP coated 10 nm particles, there was no coating-dependent difference in cytotoxicity. Furthermore, our results suggest that intracellular metal release rather than differences in cellular uptake or intracellular localization is a likely explanation for the observed differences in cytotoxicity. This study thus provides support for the so called ‘Trojan horse’ mechanism by which the particle form facilitates uptake thereby increasing the metal cellular bioavailability.

## Materials and methods

### Nanomaterials

Five types of AgNPs were investigated in this study. 10 nm OECD PVP BioPure™ Silver, 10 nm Citrate BioPure™ Silver, 40 nm Citrate BioPure™ Silver and 75 nm OECD Citrate BioPure™ Silver were purchased from NanoComposix, Inc (San Diego, CA) in the form of stock dispersions (1 mg/mL) in Milli-Q water (for the PVP coated) or aqueous 2 mM citrate (for the citrate coated). Uncoated AgNPs in the form of powder (40–50 nm) were supplied by EV NANO Technology Co Ltd, China. All particles were negative for endotoxin contamination in the limulus amebocyte lysate (LAL) test, performed as described elsewhere [51].

### Nanomaterial physico-chemical characterization

#### ***Primary characterization of AgNPs by TEM***

TEM images were acquired using a Tecnai 10 apparatus (Fei, The Netherlands) at an acceleration voltage of 100 kV and a Mega View III digital camera (Soft Imaging System, GmbH, Munchen, Germany). The particles were diluted in Milli-Q water and droplets of 3 μL were placed on TEM grids for 5 min followed by water removal with filter paper. TEM images of the uncoated Ag NPs were made using a JEOL JEM 2100F instrument operating at 200 kV.

#### ***Characterization of AgNPs in cell medium by PCCS***

The size distribution in cell medium (BEGM) was investigated using dynamic light scattering (DLS) on an instrument employing photon cross correlation spectroscopy, PCCS (NanoPhox, Sympatec, Germany). 10 μg/mL AgNPs dispersions were prepared and analyzed directly after preparation (0 h), after 4 h as well as 24 h while keeping the cuvette inside the PCCS instrument. Duplicate samples were investigated to verify the agglomeration trends but the data presented are based on single samples that were measured three times at 25°C. Data from the unique measurements was integrated to produce a single distribution with the PCCS software (Windox 5). Standard latex samples (20 ± 2 nm) (Sympatec) and blank samples were tested prior to analysis to ensure the accuracy of the measurements. The BEGM medium components resulted in a background contribution (colloids less than 5 nm) that was subtracted from the measured distribution for all AgNPs.

#### ***UV–vis spectra in cell medium***

Ultraviolet–visible (UV–vis) absorption spectra of the AgNPs dispersed in cell medium was determined on 10 μg/mL dispersions of 10 nm citrate and 10 nm PVP coated AgNPs in cell medium (BEGM) using a Jasco V-630 UV/VIS-Spectrophotometer. The absorption spectra were recorded immediately after dispersion (0 h) and after 4 as well as 24 h by keeping the cuvette inside the instrument.

### Preparation of AgNPs dispersions

The dilutions of coated AgNPs dispersions (10 nm OECD PVP BioPure™ Silver, 10 nm Citrate BioPure™ Silver, 40 nm Citrate BioPure™ Silver and 75 nm OECD Citrate BioPure™) were performed in complete cell medium prior to exposure. The 50 nm uncoated AgNPs dispersion was freshly prepared (1 mg/mL) in cell medium followed by 30 min sonication in a sonication bath on ice. Subsequent dilutions were prepared in cell medium prior to exposure.

### Cells and cell culture conditions

The normal bronchial epithelial cell line (BEAS-2B, European Collection of Cell Cultures) was used in this study. BEAS-2B cells were cultured in Bronchial epithelial cell growth medium (BEGM™, Lonza) supplemented with recombinant epidermal growth factor (EGF), hydrocortisone, insulin, bovine pituitary extract, GA-1000 (Gentamicin Sulfate and Amphotericin-B), retinoic acid, transferrin, triiodothyronine, epinephrine according to manufacturers’ instructions. No fetal calf serum was added in the cell medium. The cells were seeded in flasks and plates pre-coated with a mixture of 0.01 mg/mL fibronectin, 0.03 mg/mL bovine collagen type I, 0.01 mg/mL bovine serum albumin and 0.2% penicillin-streptomycin in BEGM additive free medium. The cells were incubated in a humidified atmosphere at 37°C, 5% CO_2_ and sub-cultured at 80% confluency.

For each experiment, BEAS-2B cells were seeded one day prior to AgNPs exposure, at an approximate density of 3 × 10^4^ cells/cm^2^ for 24 h exposure and 6 × 10^4^ cells/cm^2^ for 4 h exposure in suitable cell culture plates.

### Cellular uptake of AgNPs

#### ***Transmission electron microscopy***

BEAS-2B cells were seeded in 6 well plates and exposed to 10 μg/mL of each of the AgNP dispersions for 4 and 24 h, respectively. After exposure, cells were harvested and fixed in freshly prepared 0.1M glutaraldehyde solution, rinsed in phosphate buffer (PB) and centrifuged. The pellets were then post fixed in 2% osmium tetroxide in 0.1 M PB, pH 7.4 at 4°C for 2 h, dehydrated in ethanol followed by acetone, and embedded in LX-112 (Ladd, Burlington, Vermont, USA). Ultrathin sections (approximately 60–80 nm) were cut by a Leica ultracut UCT (Leica, Wien, Austria) and contrasted with uranyl acetate followed by lead citrate and examined with in Tecnai 12 Spirit Bio TWIN transmission electron microscope (Fei company, Eindhoven, The Netherlands) at 100 kV. Digital images were captured by using a Veleta camera (Olympus Soft Imaging Solutions, GmbH, Münster, Germany).

#### ***Atomic absorption spectroscopy***

BEAS-2B cells were seeded in 24 well plates and exposed to 10 μg/mL of each of the AgNP dispersions, in duplicates, for 4 h. After exposure the cells were thoroughly washed, harvested and counted. The total Ag concentration in solution was determined using AAS in the graphite furnace mode (Perkin Elmer Analyst 800). Calibration standards at 7.5, 15, 30, 45 μg Ag/L were prepared from a 1 g/L standard from Perkin Elmer. The calibrations curve was linear up to approx. 35 μg/L. The samples were first acidified to a pH <2 with 65% HNO3 (puriss p.a.), followed by digestion (1 mL sample + 1 mL 30 wt% H_2_O_2_ (Fluka, puriss p.a.), 3 mL 65 wt% HNO_3_ (Sigma, Suprapur, 100 μL 30 wt% HCl (Fluka, trace select) via UV treatment (Metrohm 705 UV digestor, 95°C for 2 h). As noted, 100 μL HCl was typically added as well to the digestion. This amount was, however, varied at times to confirm that all Ag was available in the form of aqueous Ag complexes. The digestion ensured that the total amount of Ag in the samples was measured using AAS. This was verified by analyzing digested samples spiked with known amounts of AgNPs. These samples yielded acceptable recoveries of the spiked Ag amount (±15% deviations). The determination limit was estimated to 5 μg/L. Triplicate readings were analyzed for each sample and control samples of known Ag concentration were analyzed in parallel generating data with the standard deviation of three independent samples and the blank value (matrix effect), if >0, subtracted. Results were expressed as the mean amount of Ag in pg/cell.

#### ***Uptake mechanisms using endocytosis inhibitors***

BEAS-2B cells were seeded in 6 well plates and pre-incubated with different pharmacological inhibitors at 37°C (amantadine 200 μM for 30 min, nystatin 25 μM for 30 min, amiloride-HCl 100 μM for 30 min, wortmannin 400 nM for 30 min, cytochalasin D 1 μM for 1 h). The selection of inhibitors was justified from their ability to selectively inhibit different pathways: amantadine blocks the clathrin-dependent endocytosis [52], nystatin disrupts caveolar structure [53], amiloride interferes with macropinocytosis [54], wortmannin reduces fluid-phase endocytosis [55] and cytochlasin D inhibits actin-dependent uptake [52]. The dose of inhibitors was selected based on previously published literature. The inhibitors were not cytotoxic at the given dose and exposure time (data not shown). For energy dependent inhibition of uptake, the cells were pre-incubated at 4°C for 30 min. Following the pre-incubations, cells were exposed to 10 μg/mL 10 nm citrate coated or 75 nm citrate coated AgNPs for 2 h in the presence of the inhibitors or at 4°C. Subsequently the cells were thoroughly washed with PBS buffer (5 times), harvested and counted using an automated cell counter (Biorad TC20). The total Ag content was determined using AAS according to the above mentioned procedure. The results were normalized according to the cell number and expressed as % of the controls (10 μg/mL 10 nm citrate coated or 75 nm citrate coated AgNPs, 2 h, without inhibitors, 37°C). Results are presented as mean ± standard deviation of 2 replicates.

### Cell viability

#### ***Lactate dehydrogenase assay***

The LDH assay is used to evaluate the degree of cellular membrane damage associated with leakage of the cytosolic LDH enzyme. The Cytotox 96® Non-Radioactive Cytotoxicity Assay Kit (Promega) was used in a 96 well plate format. The cells were exposed to the AgNP dispersions at particle doses ranging from 5 to 100 μg/mL in 100 μL for 4 and 24 h. After exposure, 50 μL of the supernatant was transferred to a new 96 well plate. The rest of the supernatant was discarded and the cells were lysed with 100 μL Triton 1% for 30 min at 37°C. 50 μL of the lysate was transferred to a new 96 well plate and 50 μL of reconstituted substrate was added to both the supernatant and the cell lysate plates. After 20 min incubation at dark conditions, reactions in both plates were terminated using 50 μL stop solution. Absorbance was measured at 495 nm using a plate reader (Tecan Infinite F200). The absorbance of the supernatant corresponds to the LDH release, whereas the sum of the absorbance of the supernatant and cell lysate corresponds to the maximum LDH release. The cell viability was calculated by dividing the LDH release to the maximum LDH release for each well. The control was set to 100% viability and the results were expressed as percentage cell viability. The experiments were performed at least three times in triplicate wells for each time point and AgNP dose. The interference of AgNPs with the LDH assay was tested in an acellular system (to test whether particles may catalyze the reaction in absence of LDH or interfere with the absorbance reading), as well as by incubating cell lysates with AgNPs before performing the assay (in order to test whether AgNPs may inhibit the LDH enzyme). The acellular interference was performed by incubating different concentrations of particles with reconstituted LDH substrate. The interference was found to be non-significant. The interference of the AgNPs with the LDH assay in terms of possible enzyme inhibition was investigated by incubating cell lysates with AgNPs (5 μg/mL and 20 μg/mL) for 0, 4 and 24 h before performing the LDH assay.

#### ***Alamar Blue assay***

The AB assay is used to assess cell viability based on the reduction potential of metabolically active cells. BEAS-2B cells were seeded in transparent 96 well plates and exposed to the AgNP dispersions at concentrations ranging from 5 to 100 μg/mL for 4 and 24 h. After exposure, 10 μL of AlamarBlue® reagent (Invitrogen) was added in each well and incubated for 2 h at 37°C. The fluorescence was measured at 560 nm excitation and 590 nm emission wavelengths using a plate reader (Tecan Infinite F200). Results were expressed as percentage cell viability versus the control. The experiments were performed at least three times in triplicate wells for each time point and AgNP dose. For the cytotoxicity of the “released fraction”, BEAS-2B cells were incubated for 24 h with the supernatant of 50 μg/mL dispersions of 10 nm PVP and citrate coated AgNPs in complete cell medium, kept at 37°C for 24 h. This experiment was performed twice in triplicate wells. Interference of the AgNPs with the assay was tested in an acellular system by incubating different doses of AgNPs (5, 10, 20, 50 μg/mL) with the AB reagent for 2 h at 37°C in 96 well plates.

### Detection of ROS production

Intracellular ROS levels were measured using the dichlorodihydrofluorescein diacetate (DCFH-DA) assay. DCFH-DA is a lipophilic cell permeable compound that is deacetylated in the cytoplasm to DCF by cellular esterases. DCF is then oxidized by radicals such as hydroxyl, peroxyl, alkoxyl, nitrate and carbonate to a fluorescent molecule (excitation 530 nm, emission 485 nm). DCF is not oxidized by hydrogen peroxide *per se* nor superoxide radical [56]. Karlsson *et al.*[24] argued that the DCF assay reflects lysosomal and mitochondrial membrane permeabilisation (that involves release of redox-active ions and cytochrome *c* that catalyzes the DCF oxidation) as the DCF accumulates in the cytosol and is unable to pass organelle membranes. BEAS-2B cells were seeded in black 96 well plates with transparent bottom and incubated with AgNPs (5, 10, 20 μg/mL) for 24 h. After exposure, cells were washed with HBSS and loaded with 20 μM DCFH-DA in HBSS (Hank’s buffered salt solution) for 30 min at 37°C. Thereafter, cells were washed with HBSS and fluorescence was recorded every 5 min over 30 min (excitation 485 nm, emission 535 nm) using a plate reader (Tecan Infinite F200) at 37°C. Tert-butyl hydroperoxide (TBP, 15 μM) was used as positive control. ROS increase was calculated as mean slope per min and normalized to the unexposed control. Results are presented as mean ± standard deviation of 4 independent experiments.

### Genotoxicity

#### ***Alkaline single-cell gel electrophoresis (comet assay)***

The comet assay is based on the microscopic detection of damaged DNA fragments of individual cells, appearing as “comets” upon cell lysis, subsequent DNA denaturation and electrophoresis. The alkaline version (PH > 13) is mostly used for the detection of single and double DNA strand breaks, DNA cross‒links, and alkali labile sites (ALSs). The comet assay is widely used to investigate genotoxicity of nanomaterials [33]. BEAS-2B cells were seeded in 24 well plates and exposed to 10 μg/mL AgNPs dispersions for 4 and 24 h. The dose was selected based on the cytotoxicity results. Cells were harvested and approximately 10^4^ cells per exposure were embedded into 0.75% low melting agarose (on 0.3% agarose pre-coated glass slides) and lysed with a freshly prepared 1% Triton lysis buffer (pH 10) for 1 h on ice at dark conditions. Alkaline unwinding was performed for 40 min on ice at dark conditions using 0.3 M NaOH (pH > 13) followed by DNA electrophoresis in the same alkaline solution for 30 min at 29 V. The slides were neutralized in 0.4 Tris Buffer for 5 min twice, dipped in deionized water and left to dry overnight. Fixation was performed in methanol for 5 min. The slides were stained with ethidium bromide and scored using a fluorescence microscope (Leica DMLB, Houston, TX) with Comet assay III software (Perceptive Instruments, Suffolk, UK). At least 50 cells were scored per sample and the results were expressed as mean percent DNA in tail. Hydrogen peroxide (25 μM) for 10 min was used a positive control. Experiments were performed at least three individual times.

#### ***Immunofluorescence staining for γH_2_AX foci***

γH_2_AX foci formation is a well-established molecular marker for DNA damage and repair. At the site of DNA double strand breaks, H_2_AX is phosphorylated at the Ser-139 residue promoting recruitment and accumulation of DNA damage response proteins [57]. BEAS-2B cells were seeded in 24 well plates on coverslips and exposed to 10 μg/mL AgNPs dispersion for 24 h. Etoposide (5 μM) was used as a positive control. After exposure, cells were fixed in 4% formaldehyde for 30 min at room temperature, followed by permeabilisation with 0.25% Triton X-100 and blocking in 3% bovine serum albumin solution. Cells were incubated with an anti-phospho-histone H_2_AX (Ser139) FITC conjugated antibody (Millipore™, 3 μg/mL) for 1 h and the coverslips were mounted with DAPI containing mounting medium. Images were acquired using a confocal laser scanning microscope (Zeiss Confocal Microscope LSM510 META) operating with LSM 5 series software. The experiments were repeated three times.

### Ag release in cell medium

The release of Ag in cell medium was determined by means of AAS. 10 μg/mL AgNPs dispersions were prepared in complete cell medium (BEGM) and kept at 37°C. After 4 and 24 h samples were centrifuged (1 h, 15 000 rpm, 0°C) and the supernatant was collected. The total Ag concentration in solution was determined using AAS in the graphite furnace mode (Perkin Elmer Analyst 800) as described in the quantification of cellular dose section. The Ag release was also measured in ALF (artificial lysosomal fluid). The artificial lysosomal fluid has a pH of 4.5 and is intended to mimic the lysosomal acidic environment. ALF composition in g/L follows: MgCl_2_ 0.050, NaCl 3.21, Na_2_HPO_4_ 0.071, Na_2_SO_4_ 0.039, CaCl_2_ · 2H_2_O 0.128, C_6_H_5_Na_3_O_7_ · 2H_2_O 0.077, NaOH 6.00, C_6_H_8_O_7_ 20.8, H_2_NCH_2_COOH 0.059, C_4_H_4_O_6_Na_2_ · 2H_2_O 0.090, C_3_H_5_NaO_3_ 0.085, C_3_H_3_O_3_Na 0.086 [58,59]. 10 μg/mL AgNPs dispersions were prepared in ALF and kept at 37°C. After 4 and 24 h samples were centrifuged (1 h, 15 000 rpm, 0°C), the supernatant was collected and analyzed by AAS according to the previously mentioned protocol.

### Statistical analysis

Data was analyzed in GraphPad Prism (version 5.02) by one-way or two-way analysis of variance (ANOVA) followed by Dunnett’s multiple comparison and Bonferroni post-tests, respectively. P-values lower than 0.05 were considered statistically significant. The error bars represent standard deviation of the mean.

## Abbreviations

AAS: Atomic absorption spectroscopy; AB: Alamar Blue; AgNPs: Silver nanoparticles; BEGM: Bronchial epithelial cell growth medium; DCFH-DA: Dichlorodihydrofluorescein diacetate; LDH: Lactate dehydrogenase; OECD: Organization for economic co-operation and development; PCCS: Photon cross correlation spectroscopy; ROS: Reactive oxygen species.

## Competing interests

The authors declare that they have no competing interests.

## Authors’ contribution

ARG participated in the design of the study, carried out all the cellular experiments, performed the statistical analysis, data interpretation and drafted as well as finalized the manuscript. SS performed most of the PCCS and AAS analyses, was involved in interpretation of these results as well as in the preparation of the manuscript and figures of the particle characterization. IOW supervised the particle characterization, AAS analyses and their interpretations and was involved in manuscript writing. BF participated in the design of the study and was involved in data interpretation and manuscript writing. HLK initiated the study, supervised the cellular experiments and data interpretation and was involved in the manuscript preparation and writing. All authors read and approved the final manuscript.

## Supplementary Material

Additional file 1: Table S1 The particle size distribution in cell medium (BEGM) by volume and the scattered light intensity determined by PCCS. The particle size distribution by volume corresponds to 10% (d0.1), 50% (d0.5) and 90% (d0.9) and the scattered light intensity of the measurement depends on the size and concentration of the particles in solution. Click here for file

Additional file 2: Figure S1 Cell viability of BEAS-2B cells after 4 h exposure to AgNPs. Cell viability of BEAS-2B cells after 4 h exposure to AgNPs (5–50 μg/mL) was assessed using AB assay **(A)** and LDH assay **(B)**. Results are presented as mean ± standard deviation of 3 independent experiments. Click here for file

Additional file 3: Figure S2 Interference of AgNPs with the Alamar Blue assay. Different concentrations (5–50 μg/mL) of AgNPs dispersions in BEGM cell medium were incubated with the AB reagent for 2 h at 37°C in 96 well plates and fluorescence was recorded (Ex560/Em590). A cellular system with 80% confluent BEAS-2B cells was used as a reference. For all the AgNPs there was a slight dose dependent increase in fluorescence (Ex560/Em590). However this increase is not significant when compared to the cellular systems (25 fold higher) and is unlikely to interfere with the results. **Figure S3.** Interference of AgNPs with the LDH assay. BEAS-2B cells were seeded in 96 well plates and lysed the following day with he the same lysis agent as in the LDH protocol. The lysate was incubated with AgNPs (5 μg/mL and 20 μg/mL) for 0, 4 and 24 h before performing the LDH assay. The results show that the enzyme activity decreased over time for all samples. At timepoint 0 there was no major difference between samples with no signs of LDH enzyme inhibition. After 4 h incubation there was a decrease in enzyme activity for the 10 nm AgNPs and also for the 75 nm AgNPs at the highest concentration (20 μg/mL). After 24 h, a dose dependent decrease in LDH activity was observed for the 10 nm AgNPs, especially for the citrate coated ones, and to some extent also for the 40 nm coated particles at the highest dose. Click here for file

Additional file 4: Figure S4 ROS levels in BEAS-2B cells during 4 h exposure to AgNPs. ROS formation after exposure to AgNPs was investigated using the DCFH-DA assay. Cells were incubated with AgNPs (5, 10, 20 μg/mL) or tert-butyl hydroperoxide (TBP, 200 μM, positive control) for 4 h with readings (excitation 485 nm, emission 535 nm) performed every 30 min. ROS induction was expressed as mean slope per hour and normalized to the unexposed control. Results are presented as mean ± standard deviation of 3 independent experiments. Click here for file

Additional file 5: Figure S5 TEM images of BEAS-2B cells after 4 h exposure to AgNPs. TEM images of untreated BEAS-2B cells showed no morphological changes **(A, a)**. After 4 h exposure to 10 μg/mL 10 nm citrate coated **(B, b)**, 10 nm PVP coated **(C, c)**, 40 nm citrate coated **(D, d)**, 75 nm citrate coated **(E, e)** and 50 nm uncoated **(F, f)** AgNPs, there was clear particle localization within endo-lysosomal vesicles (black arrows). Click here for file

Additional file 6: Figure S6 Ag release in artificial lysosomal fluid (ALF). The amount of Ag release in ALF solution over 4 and 24 h at 37°C was quantified by means of AAS and expressed as the percentage of the total amount of added Ag (10 μg/mL). The overall amount of Ag released and measured in solution was very low (less than 2%), considerably lower than the release in cell medium. This was likely related to increased agglomeration together with complexation and sedimentation of silver species (such as AgCl) followed by removal upon particle separation. Click here for file
