# Supplementary material for: Size-dependent cytotoxicity of silver nanoparticles in human lung cells: the role of cellular uptake, agglomeration and Ag release
Source: Part Fibre Toxicol. 2014 Feb 17;11:11. doi: 10.1186/1743-8977-11-11 (PMC3933429; doi:10.1186/1743-8977-11-11)
Supplement: Additional file 1: Table S1 — The particle size distribution in cell medium (BEGM) by volume and the scattered light intensity determined by PCCS. The particle size distribution by volume corresponds to 10% (d0.1), 50% (d0.5) and 90% (d0.9) and the scattered light intensity of the measurement depends on the size and concentration of the particles in solution. [file 1743-8977-11-11-S1.pdf]

Additional file 1. AgNPs size distribution in cell medium by PCCS

Table S1: The particle size distribution in cell medium (BEGM) by volume and the scattered light intensity determined by PCCS. The particle size distribution by volume corresponds to 10% ($d_{0.1}$), 50% ($d_{0.5}$) and 90% ($d_{0.9}$) and the scattered light intensity of the measurement depends on the size and concentration of the particles in solution.

Sample	Time point (h)	$d_{0.1}$ [nm] (volume)	$d_{0.5}$ [nm] (volume)	$d_{0.9}$ [nm] (volume)	Scattered light intensity [kcps]
BEGM (blank)	-	1	2	2	3
10 nm citrate coated	0	2	7	20	730
	4	2	7	3430	410
	24	2	11	840	63
10 nm PVP coated	0	1	2	3	970
	4	1	3	5	1000
	24	2	6	23	709
40 nm citrate coated	0	7	17	350	3200
	4	3	24	3900	1400
	24	19	34	300	800
75 nm citrate coated	0	2	4	14	2790
	4	2	74	380	2700
	24	4	19	380	1100
50 nm uncoated	0	1	3	7	860
	4	2	89	520	500
	24	2	9	660	90
