# Supplementary material for: Size-dependent cytotoxicity of silver nanoparticles in human lung cells: the role of cellular uptake, agglomeration and Ag release
Source: Part Fibre Toxicol. 2014 Feb 17;11:11. doi: 10.1186/1743-8977-11-11 (PMC3933429; doi:10.1186/1743-8977-11-11)
Supplement: Additional file 2: Figure S1 — Cell viability of BEAS-2B cells after 4 h exposure to AgNPs. Cell viability of BEAS-2B cells after 4 h exposure to AgNPs (5–50 μg/mL) was assessed using AB assay (A) and LDH assay (B). Results are presented as mean ± standard deviation of 3 independent experiments. [file 1743-8977-11-11-S2.pdf]

Additional file 2. Cell viability of BEAS-2B cells after 4 h exposure to AgNPs

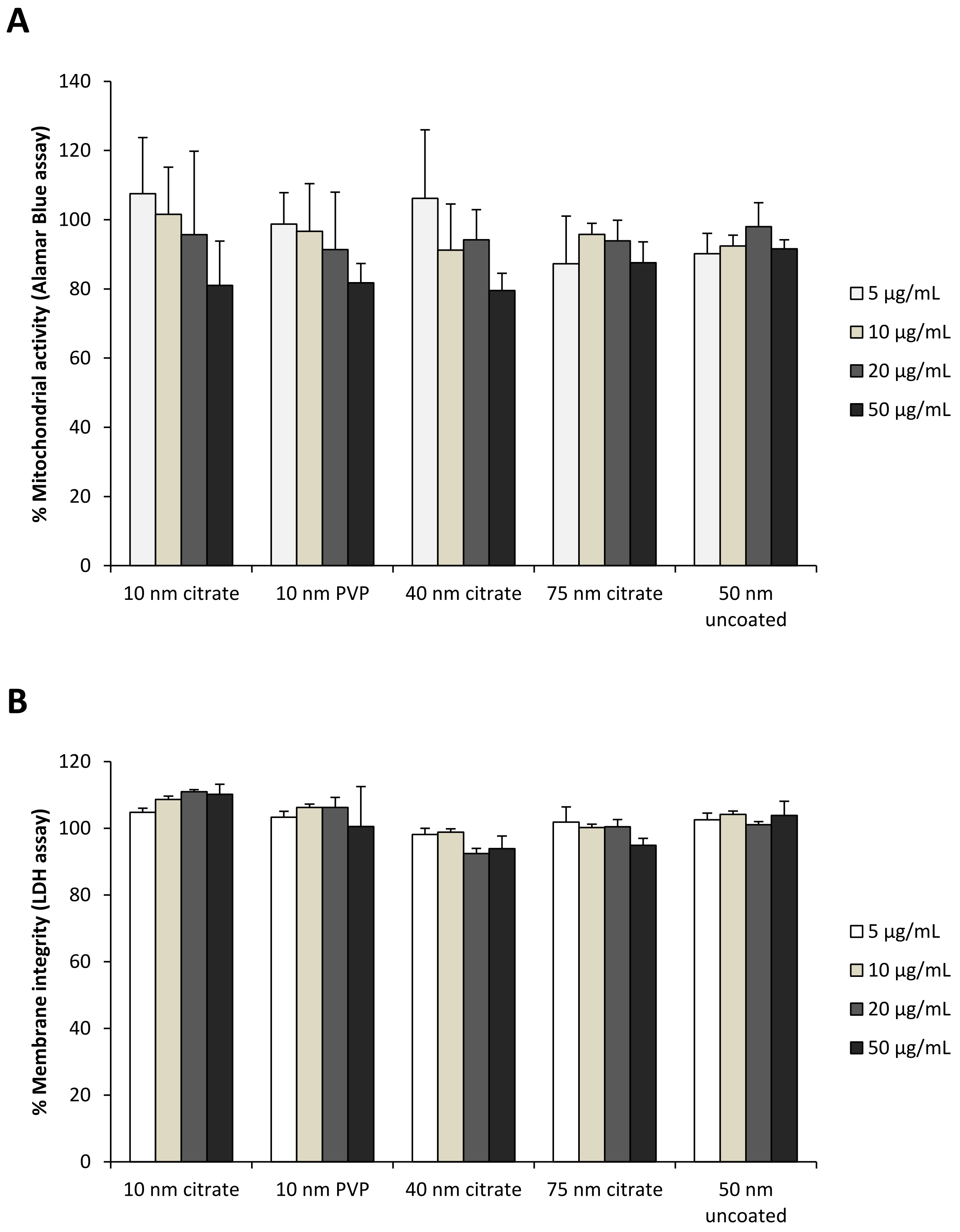

Figure S1. Cell viability of BEAS-2B cells after 4 h exposure to AgNPs. Cell viability of BEAS-2B cells after 4 h exposure to AgNPs (5 – 50 µg/mL) was assessed using AB assay (A) and LDH assay (B). Results are presented as mean ± standard deviation of 3 independent experiments.
