# Supplementary material for: Size-dependent cytotoxicity of silver nanoparticles in human lung cells: the role of cellular uptake, agglomeration and Ag release
Source: Part Fibre Toxicol. 2014 Feb 17;11:11. doi: 10.1186/1743-8977-11-11 (PMC3933429; doi:10.1186/1743-8977-11-11)
Supplement: Additional file 3: Figure S2 — Interference of AgNPs with the Alamar Blue assay. Different concentrations (5–50 μg/mL) of AgNPs dispersions in BEGM cell medium were incubated with the AB reagent for 2 h at 37°C in 96 well plates and fluorescence was recorded (Ex560/Em590). A cellular system with 80% confluent BEAS-2B cells was used as a reference. For all the AgNPs there was a slight dose dependent increase in fluorescence (Ex560/Em590). However this increase is not significant when compared to the cellular systems (25 fold higher) and is unlikely to interfere with the results. Figure S3. Interference of AgNPs with the LDH assay. BEAS-2B cells were seeded in 96 well plates and lysed the following day with he the same lysis agent as in the LDH protocol. The lysate was incubated with AgNPs (5 μg/mL and 20 μg/mL) for 0, 4 and 24 h before performing the LDH assay. The results show that the enzyme activity decreased over time for all samples. At timepoint 0 there was no major difference between samples with no signs of LDH enzyme inhibition. After 4 h incubation there was a decrease in enzyme activity for the 10 nm AgNPs and also for the 75 nm AgNPs at the highest concentration (20 μg/mL). After 24 h, a dose dependent decrease in LDH activity was observed for the 10 nm AgNPs, especially for the citrate coated ones, and to some extent also for the 40 nm coated particles at the highest dose. [file 1743-8977-11-11-S3.pdf]

Additional file 3. Interference of the AgNPs with the Alamar Blue and LDH assays

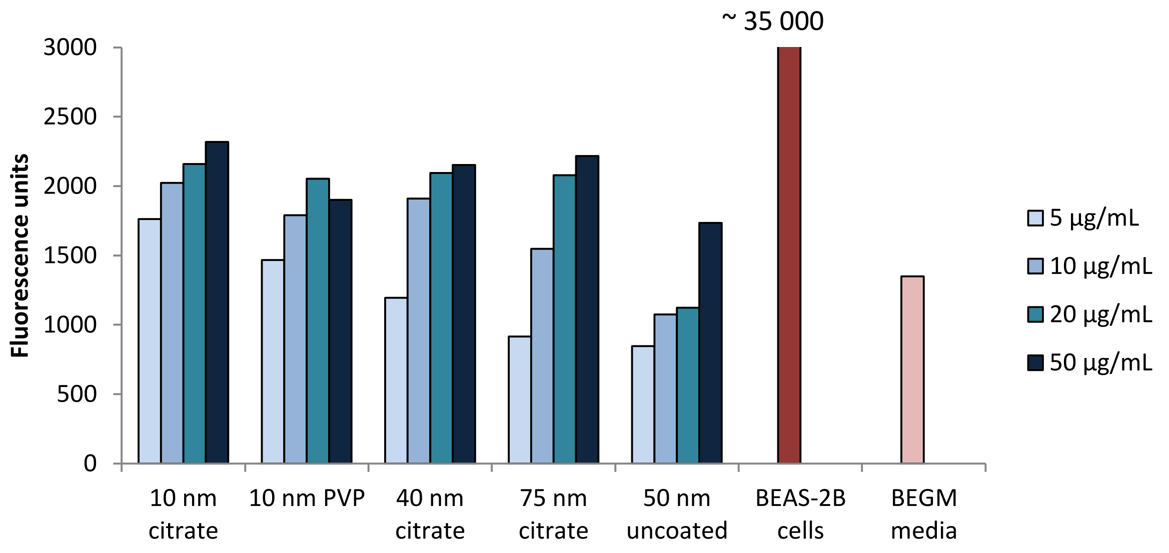

Figure S2. Interference of AgNPs with the Alamar Blue assay. Different concentrations (5 - 50 µg/mL) of AgNPs dispersions in BEGM cell medium were incubated with the AB reagent for 2 h at 37°C in 96 well plates and fluorescence was recorded (Ex560/Em590). A cellular system with 80% confluent BEAS-2B cells was used as a reference. For all the AgNPs there was a slight dose dependent increase in fluorescence (Ex560/Em590). However this increase is not significant when compared to the cellular systems (25 fold higher) and is unlikely to interfere with the results.

Additional file 3. Interference of the AgNPs with the Alamar Blue and LDH assays

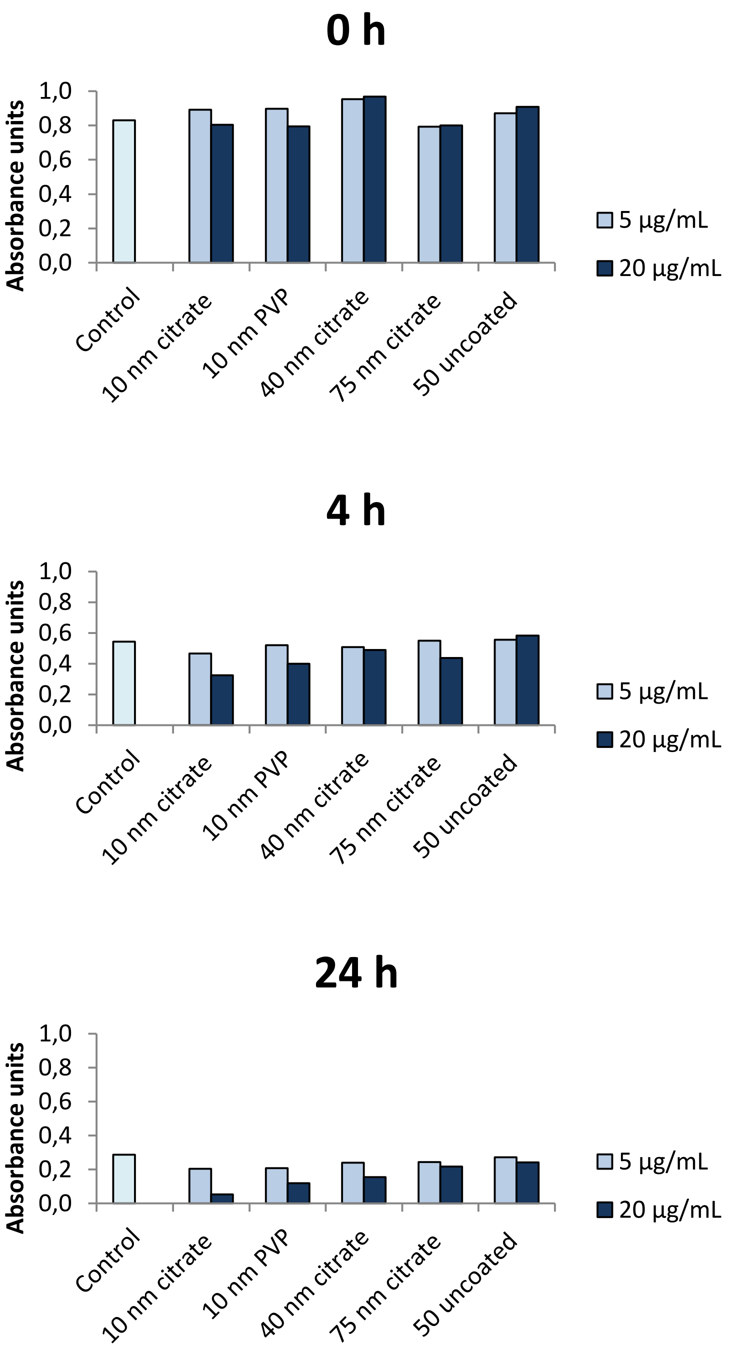

Figure S3. Interference of AgNPs with the LDH assay. BEAS-2B cells were seeded in 96 well plates and lysed the following day with he the same lysis agent as in the LDH protocol. The lysate was incubated with AgNPs (5 µg/mL and 20 µg/mL) for 0, 4 and 24 h before performing the LDH assay. The results show that the enzyme activity decreased over time for all samples. At timepoint 0 there was no major difference between samples with no signs of LDH enzyme inhibition. After 4 h incubation there was a decrease in enzyme activity for the 10 nm AgNPs and also for the 75 nm AgNPs at the highest concentration (20 µg/mL). After 24 h, a dose dependent decrease in LDH activity was observed for the 10 nm AgNPs, especially for the citrate coated ones, and to some extent also for the 40 nm coated particles at the highest dose
