# Supplementary material for: Size-dependent cytotoxicity of silver nanoparticles in human lung cells: the role of cellular uptake, agglomeration and Ag release
Source: Part Fibre Toxicol. 2014 Feb 17;11:11. doi: 10.1186/1743-8977-11-11 (PMC3933429; doi:10.1186/1743-8977-11-11)
Supplement: Additional file 4: Figure S4 — ROS levels in BEAS-2B cells during 4 h exposure to AgNPs. ROS formation after exposure to AgNPs was investigated using the DCFH-DA assay. Cells were incubated with AgNPs (5, 10, 20 μg/mL) or tert-butyl hydroperoxide (TBP, 200 μM, positive control) for 4 h with readings (excitation 485 nm, emission 535 nm) performed every 30 min. ROS induction was expressed as mean slope per hour and normalized to the unexposed control. Results are presented as mean ± standard deviation of 3 independent experiments. [file 1743-8977-11-11-S4.pdf]

Additional file 4. ROS levels during 4 h exposure to AgNPs

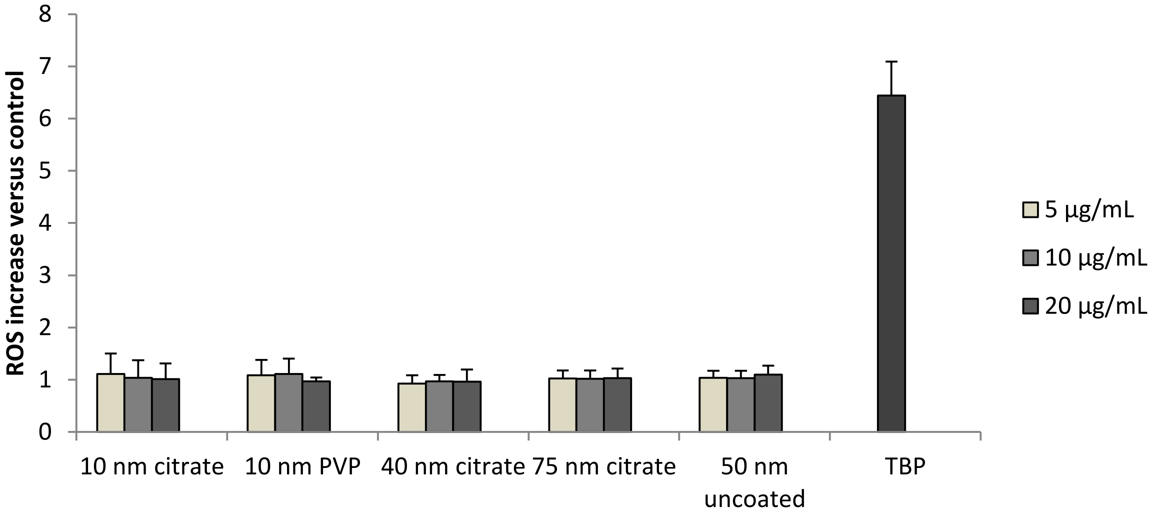

Figure S4. ROS levels in BEAS-2B cells during 4 h exposure to AgNPs. ROS formation after exposure to AgNPs was investigated using the DCFH-DA assay. Cells were incubated with AgNPs (5, 10, 20 µg/mL) or tert-butyl hydroperoxide (TBP, 200 µM, positive control) for 4 h with readings (excitation 485 nm, emission 535 nm) performed every 30 min. ROS induction was expressed as mean slope per hour and normalized to the unexposed control. Results are presented as mean ± standard deviation of 3 independent experiments.
