# Supplementary material for: Size-dependent cytotoxicity of silver nanoparticles in human lung cells: the role of cellular uptake, agglomeration and Ag release
Source: Part Fibre Toxicol. 2014 Feb 17;11:11. doi: 10.1186/1743-8977-11-11 (PMC3933429; doi:10.1186/1743-8977-11-11)
Supplement: Additional file 5: Figure S5 — TEM images of BEAS-2B cells after 4 h exposure to AgNPs. TEM images of untreated BEAS-2B cells showed no morphological changes (A, a). After 4 h exposure to 10 μg/mL 10 nm citrate coated (B, b), 10 nm PVP coated (C, c), 40 nm citrate coated (D, d), 75 nm citrate coated (E, e) and 50 nm uncoated (F, f) AgNPs, there was clear particle localization within endo-lysosomal vesicles (black arrows). [file 1743-8977-11-11-S5.pdf]

Additional file 5. Cellular uptake and localization of AgNPs after 4 h exposure in BEAS-2B cells

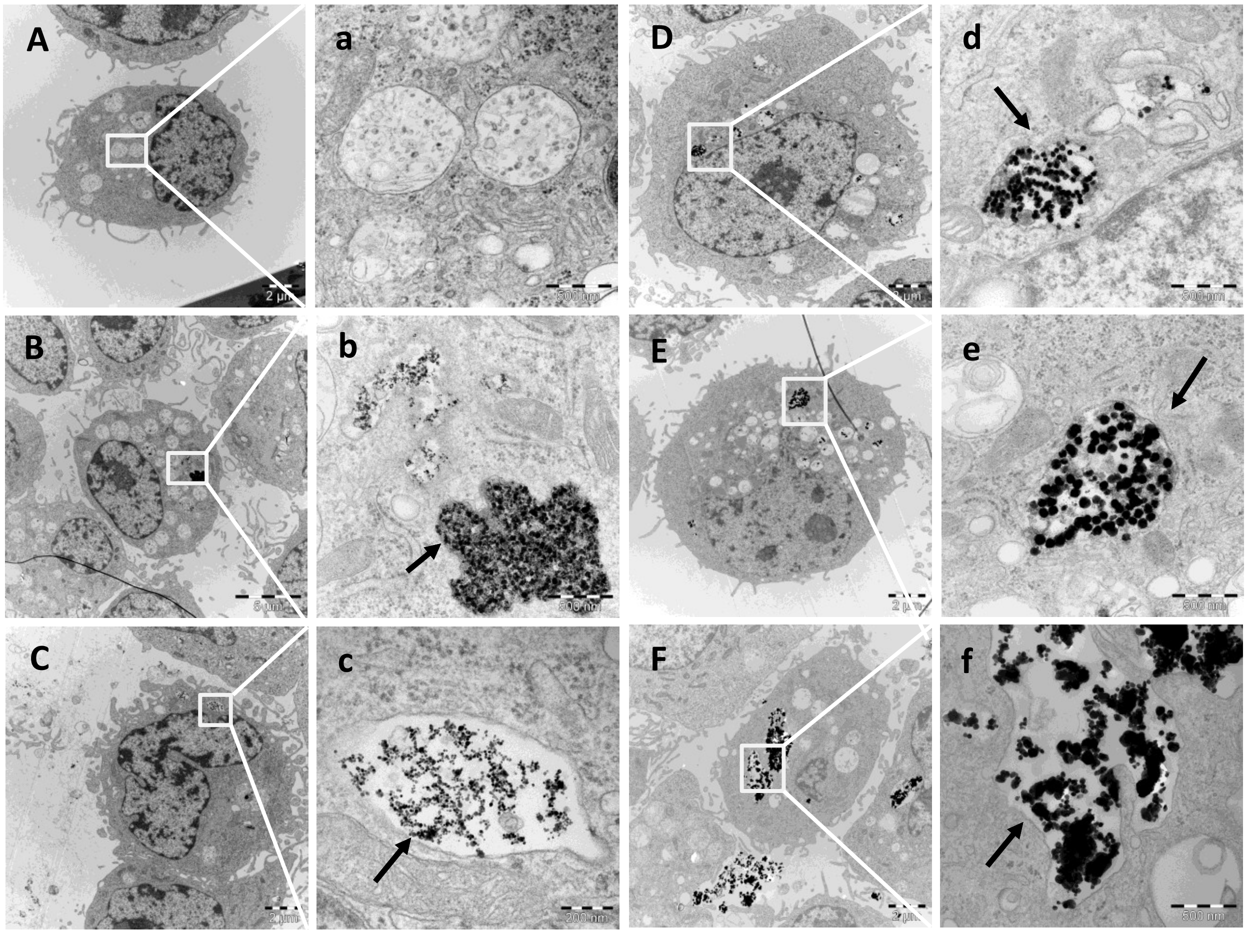

Figure S5. TEM images of BEAS-2B cells after 4 h exposure to AgNPs. TEM images of untreated BEAS-2B cells showed no morphological changes (A, a). After 4 h exposure to 10 µg/mL 10 nm citrate coated (B, b), 10 nm PVP coated (C, c), 40 nm citrate coated (D, d), 75 nm citrate coated (E, e) and 50 nm uncoated (F, f) AgNPs, there was clear particle localization within endo-lysosomal vesicles (black arrows).
