# Supplementary material for: Size-dependent cytotoxicity of silver nanoparticles in human lung cells: the role of cellular uptake, agglomeration and Ag release
Source: Part Fibre Toxicol. 2014 Feb 17;11:11. doi: 10.1186/1743-8977-11-11 (PMC3933429; doi:10.1186/1743-8977-11-11)
Supplement: Additional file 6: Figure S6 — Ag release in artificial lysosomal fluid (ALF). The amount of Ag release in ALF solution over 4 and 24 h at 37°C was quantified by means of AAS and expressed as the percentage of the total amount of added Ag (10 μg/mL). The overall amount of Ag released and measured in solution was very low (less than 2%), considerably lower than the release in cell medium. This was likely related to increased agglomeration together with complexation and sedimentation of silver species (such as AgCl) followed by removal upon particle separation. [file 1743-8977-11-11-S6.pdf]

Additional file 6. Ag release in artificial lysosomal fluid

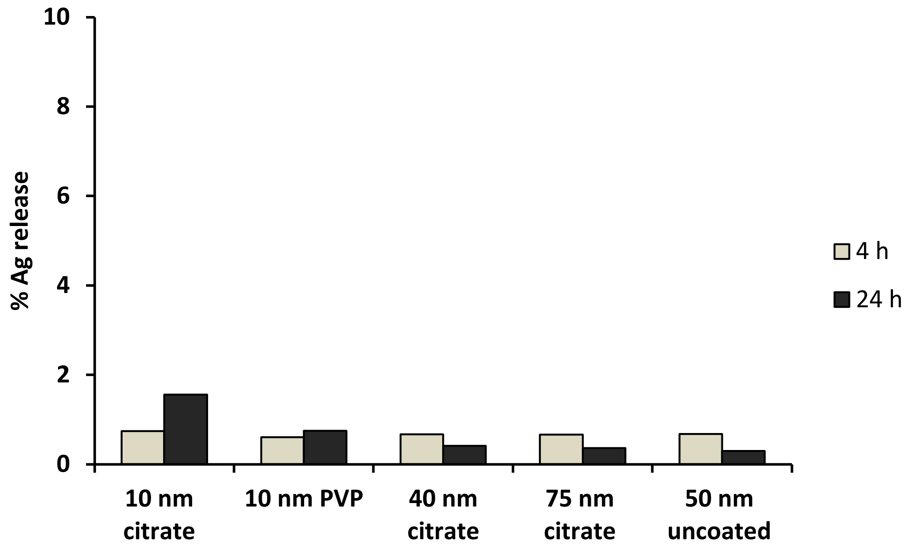

Figure S6. Ag release in artificial lysosomal fluid (ALF). The amount of Ag release in ALF solution over 4 and 24 h at 37°C was quantified by means of AAS and expressed as the percentage of the total amount of added Ag (10 µg/mL). The overall amount of Ag released and measured in solution was very low (less than 2%), considerably lower than the release in cell medium. This was likely related to increased agglomeration together with complexation and sedimentation of silver species (such as AgCl) followed by removal upon particle separation.
